# Strategies to meet marital intimacy needs in women infected with coronavirus 2019: A framework qualitative content analysis research

**DOI:** 10.18502/ijrm.v21i9.14399

**Published:** 2023-10-30

**Authors:** Tahmineh Farajkhoda, Mahmood Kamali Zarch, Saeedeh Najafihedeshi

**Affiliations:** ^1^Research Center for Nursing and Midwifery Care, Non-Communicable Diseases Institute, Midwifery Department, School of Nursing and Midwifery, Shahid Sadoughi University of Medical Sciences, Yazd, Iran.; ^2^Department of Psychology, Payame Noor University, Tehran, Iran.; ^3^Student Research Committee, School of Nursing and Midwifery, Shahid Beheshti University of Medical Sciences, Tehran, Iran.

**Keywords:** Counseling, Intimacy, Qualitative research, Sexual health, Women.

## Abstract

**Background:**

Separating women with the coronavirus 2019 from family can affect marital life. Considering psychological vulnerability of women for anxiety and depression disorders, these women may experience more stress due to the loss of work at this time or special reproductive health conditions such as pregnancy, having a baby, or other medical conditions.

**Objective:**

Considering a limited number of qualitative studies on the need for marital intimacy, this study was conducted to meet marital intimacy needs in women with coronavirus 2019.

**Materials and Methods:**

Framework qualitative content analysis was conducted through participation of 13 key informants (sex therapist, couple therapist, psychologist, and midwifery counselor) and 18 infected women from Yazd, Iran in July 2020 who were selected purposefully and interviewed through the semi-structured in-depth interviews.

**Results:**

3 main categories were: 1) mutual resilience (subcategories: 1. coping strategies; 2. value of marital life; 3. compassionate conflict resolving). 2) skillful relationship (subcategories: 1. assertiveness in sexual expression; 2. self-efficacy in the distance physical connectivity). 3) synergy (subcategories: 1. reframing spirituality closeness; 2. empowering aesthetic creativity; 3. management of family function, and 4. prioritizing).

**Conclusion:**

Findings revealed sex and couple therapists, health providers, and policymakers should emphasize on using new forms of digital communication in these couples. Teaching skills that increase partners' creativity and empathy, enable women to fulfill their mental, sexual and reproductive health needs, and lead to more partners responsibility and loyalty, and maintenance of family function. In times of crisis, counseling should be considered in women treatment programs and care guidelines.

## 1. Introduction

The significant effect of the coronavirus is related to a gender-based vulnerability in women. They are more psychologically vulnerable to anxiety and depressive disorders. Infected women may experience additional stress due to financial problems and their reproductive health-specific conditions like pregnancy, having a newborn/baby, medical conditions, having the role of caregiver for family members, and consequences of family members' future well-being (1, 2). According to the United Nations Women's report in January 2021, the outbreak of Coronavirus 2019 (COVID-19) has increased the rate of intimate partner violence globally (1, 3). Recent studies have been conducted to establish the negative impact of COVID-19 on sexual and marital relationships (1, 4, 5). There are a few reports on marital intimacy needs in COVID-19-infected partners who must isolate themselves from their noninfected partners and family members at home (1, 6). All dimensions of marital intimacy can be affected in COVID-19-infected women (7, 8). They must avoid sexual intercourse and close physical and respiratory contact until they get better (7). Although the isolation period is not very long, avoiding usual contact with the partner, family members, and others can have significant psychological and marital intimacy consequences. In addition, even after the end of the transmission period, some women or their partners refuse to reestablish marital, physical, and sexual relationships due to fear of transmission to their partners or fear of being infected by their infected partners (5, 7-9).

Marital intimacy means sharing intimate marital experiences in order to fulfill the psychological and emotional needs such as attention, trust, closeness, care, responsiveness, and security (10, 11). Bagarozzi's marital intimacy needs theory defines marital intimacy in 9 dimensions, emotional, cognitive, rational, sexual, physical, spiritual, aesthetic, social recreational, and temporal (10). In a qualitative study, marital intimacy-promoting factors were family, shared time/length of marital relationship, reciprocity in self-sacrifice, gratitude, new shared activity, parenthood, collaborative social networks, and religion (12). The main question is how COVID-19 affects marital intimacy? In one study a preliminary framework was introduced regarding the impact of COVID-19 on sexual health (4). In another research, a correlation between sexual behavior change, family function, and male-female intimacy was demonstrated among adults during COVID-19 epidemic (6). The sexual dimensions in COVID-19 crisis were explained in one study; in a review article intimacy and sexual well-being challenges were reported during COVID-19 outbreak. The conflicts of the romantic relationship was discussed during COVID-19 outbreak by investigators, and one article addressed marital stress in COVID-19 outbreak (2, 5, 7, 9). During the COVID-19 epidemic, all dimensions of marital intimacy (not only sexual domain) should be considered in different domains of the physical, psychological, sexual, and social health of couples, such as their quality of life, marital relationship, and well-being to provide appropriate response to meet couples' marital intimacy needs (2, 6, 9, 10). When marital life encounters any transition (like adversity due to COVID-19), it needs to readjust itself (12).

In this regard, many qualitative studies are needed to understand couples' experiences during this pandemic (5). Qualitative research can provide more practical and scientific preparation for counselors, psychologists, couple therapists, physicians, and other healthcare providers who work in the field of marital intimacy and couples who have been involved. These findings can be helpful to the present time and the outbreak of other similar diseases in the future. Framework qualitative content analysis is an appropriate method to reveal unknown needs for having marital intimacy during home isolation within Bragozzi's 9 dimensions of marital intimacy needs theory, not just its sexual dimension (10). Qualitative content analysis is becoming increasingly popular for managing and analyzing qualitative data by providing clear steps to follow. It produces highly structured outputs of summarized data (13).

The study question is what strategies can help COVID-19-infected women to meet their marital intimacy needs? The current research aimed strategies to meet marital intimacy needs in women infected with COVID-19.

## 2. Materials and Methods

### Type of study

This qualitative framework content analysis was conducted from July 2020 to April 2021 in Yazd, Iran, to explain marital intimacy needs in women infected with COVID-19. The naturalism approach was chosen for a qualitative study to better explain the phenomenon in its real and cultural context and describe it as experienced (14). Qualitative studies are the most appropriate method for research on topics whose various dimensions are not well studied and need further investigation, like COVID-19 (15). The current study used 9 dimensions of Bagarozzi's theory of marital intimacy needs (10). In the current study, the main reason for choosing a framework of qualitative content analysis was selecting an efficient approach for managing qualitative data related to health and explaining the phenomenon with multiple dimensions, such as marital intimacy. In addition, this approach provides a framework for conducting interviews within this framework for scientific categorization (13).

### Sampling, sample size, and participants

Sampling was done in a purposeful method to achieve data saturation. 13 key informants (sex therapists, psychologists, couple therapists, and counselors in midwifery) and 18 married infected women with COVID-19 in home isolation participated in the research.

Key informants are experts who are valuable sources of information and knowledge about the phenomenon. They are knowledgeable, willing to participate, communicative, and have enough skills (15). Maximum sample variation used was based on the selection of women. They were selected with various demographic characteristics such as age, education, occupation, fertility history, rural and urban community in women's inclusion criteria who were infected with COVID-19 (according to the national protocol of diagnosis and treatment of COVID-19 established by the Ministry of Health and Medical Education of Iran), the need for home isolation in a separate room, and living with a spouse. In year 2020, routine vaccination for COVID-19 was not available and accessible worldwide. In addition, to achieve rich data, key informants were invited to participate in this study in various related disciplines from different universities of Iran with different ages, genders, and work length experiences in the field (at least 5 yr work length experience as inclusion criterion).

### Study setting

Due to the COVID-19 outbreak, women were invited to participate in this research by a phone call in the healthcare centers of Yazd. Key informants were invited to participate in the study via online WhatsApp voice calls from various universities of Iran.

### Data collection tool

Semi-structured interviews with open-ended questions were used to interview all women and key informants. The first author conducted all the interviews. Questions were prepared based on dimensions of marital intimacy from Bragozzi's theory of marital intimacy needs (10). Women answered questions such as “what were your experiences when you were infected with COVID-19?” “What problems did you experience in terms of different dimensions of marital intimacy during the disease?” “How did you manage marital intimacy needs during the disease?” and “what did you need to overcome your marital intimacy problems? Moreover, what were your marital intimacy expectations that your spouse could meet?” Key informants answered questions like “what are the needs of marital intimacy in women who were infected with COVID-19, based on the dimensions of marital intimacy needs theory?” and “what strategies do you suggest to meet the needs of marital intimacy based on the dimensions of marital intimacy needs theory?” The interviews lasted 45-60 min.

### Data collection method

The first author conducted all interviews via voice call. After ending the interview, women who had participated in the interviews received one free of-charge session for counseling regarding marital intimacy by the first author as an appreciation of their participation in the study. The first author interviewed key informants via WhatsApp voice call. All semi-structured interviews were developed and conducted by the first author.

### Study rigor

4 Lincoln and Guba criteria, credibility, dependability, confirmability, and transferability, were used to achieve trustworthiness in this research (14). Our researchers' team performed credibility via using prolonged engagement and continuous observation, member checking, search for negative case analysis, and peer debriefing. A thick description of more details about the participants and the environment's characteristics were provided by the researchers to ensure transferability. Dependability was done by documenting all activities so that other people could follow them as external audits. Confirmability was obtained by using confirmability audit, audit trail, and reflexivity. 32-item COREQ (Consolidated criteria for reporting qualitative research) checklist criteria (16) was used to evaluate the quality of the current research and report it.

### Ethical considerations

The Ethics Committee of Shahid Sadoughi University of Medical Sciences, Yazd, Iran approved the study protocol (Code: IR.SSU.REC.1399.059). Informed consent was obtained from all participants, by the first author.

### Statistical analysis

The whole of the transcripted interviews and memos were considered as an analysis unit. A 7-step framework qualitative content analysis method was used for qualitative data analysis. Framework qualitative content analysis is a flexible method in the analysis process and allows the researcher to analyze the data after complete or during the data collection process (13, 17). In the current study, the researcher analyzed the data during data collection manually. The 7-step analysis process included 1) familiarity with the texts; 2) implementation and copying; 3) data organization; 4) a thematic framework; 5) indexing; 6) tabulation; and 7) final interpretation. Data collection and analysis were finished when sampling was completed, and new data did not produce new insights.

The first author coded the data in cooperation with the second and third authors. For more study validity, each author coded the raw data individually into categories and subcategories. The authors were involved in the coding revision, which led to an interpretative step; the continuous codification, interpretation, and recodification of data led to a final category. After they reached a consensus and agreement, the participants' answers formed the categories and subcategories and led to the conceptual model.

## 3. Results

**Figure 1:**
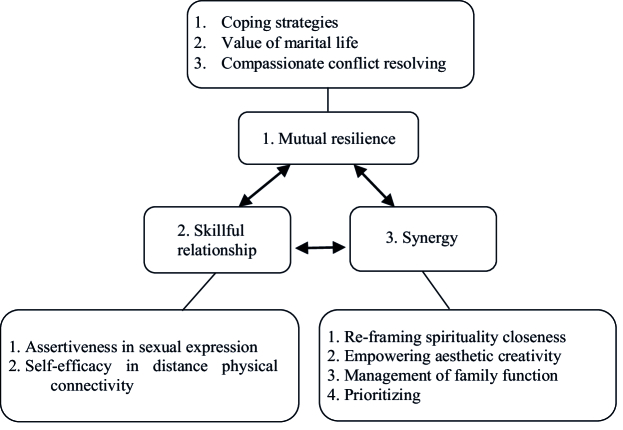
Strategies to meet marital intimacy needs of COVID-19-infected women.

The demographic characteristics of key informants and women who participated in this study have been shown in table I. After omitting unrelated codes, primary codes were formulated into 3 main categories: 1) Mutual resilience (subcategories: 1. coping strategies; 2. value of marital life; 3. compassionate conflict resolving). 2) Skillful relationship (subcategories: 1. assertiveness in sexual expression; 2. self-efficacy in distance physical connectivity). 3) Synergy (subcategories: 1. re-framing spirituality closeness; 2. empowering aesthetic creativity; 3. management of family function; and 4. prioritizing) (Table II). Participants' comprehensive answers formed strategies to meet marital intimacy needs (Figure 1).

### Mutual resilience

The main category of mutual resilience integrated participants' answers that focused on the need to accept the critical condition and deal with it logically. Couples need to use appropriate strategies (e.g., subcategory 1.1. coping strategies). Mutual resilience further aggregates concepts like thinking reconstruction due to marital life (e.g., subcategory 1.2. value of marital life). In addition, it pertained to the need to solve the marital problems that emerged from COVID-19 (e.g., subcategory 1.3. compassionate conflict resolving).

### Skillful relationship

The main category of the skillful relationship integrates answers emphasizing the need for skillful behaviors in different dimensions. Participants believed that the need for sexual relationship expression is particularly important (e.g., subcategory 2.1. assertiveness in the sexual expression). The need for physical relationships was another stated issue regarding skillful relationships in this regard by participants (e.g., subcategory 2.2. self-efficacy in distance physical connectivity).

### Synergy

The third main category, synergy, referred to other marital intimacy needs like spiritual, aesthetic, social-recreational, and temporal that should be expressed innovatively. Participants highlighted the role of spirituality as a relief for couples suffering (e.g., subcategory 3.1. re-framing spirituality closeness). It also included a subcategory targeting aesthetic matters to answer couples' aesthetic creativity needs (e.g., subcategory 3.2. empowering aesthetic creativity). Synergy further aggregated concepts like family function role (e.g., subcategory 3.3. management of family function). Synergy additionally included prioritizing in couple's time devoted for each other (e.g., subcategory 3.4. prioritizing).

**Table 1:** Demographic characteristics of key informants and women who participated in this research


**Variables**			**Frequency/range**
**Key informants**			
	Age (yr)*		31-58
	Work experience (yr)*		5-27
	Discipline**		
	**Sex therapist**	6
	**Couple therapist**	1
	**Psychologist**	2
	**Midwifery counselor**	4
**Women participated in this study**			
	Age (yr)*		18-57
	Duration of marriage (yr)*		1-38
	Job**		
	**Housewife**	8
	**Employee**	4
	**Home jobs**	3
	**Worker**	3
*Data presented as min-max. **Data presented as frequency			

**Table 2:** Categories, subcategories, and codes


**Categories**			**Examples of participants narrations**
**1. Mutual resilience**			
	**1.1. Coping strategies**		
	**1.1.1. Empathetic support**	“My spouse said to me: I'm here to help you”. (A 27-yr-old pregnant woman) “If couples show empathic behaviors, they can cope with this difficult situation”. (41-yr-old couple therapist)
	**1.1.2. Emotion regulation**	“Our poor economic status cause unpleasant emotions such as fault, sadness, anger, blame and fear of family contamination. I should do something”. (A 36-yr-old female worker with 3 children)
	**1.1.3. Miracle of positive** **thoughts**	“Replacement of negative thoughts and ruminations with positive ones acts as a miracle”. (52-yr-old psychologist)
	**1.2. Value of marital life**		
	**1.2.1. Marital commitment**	“Couples need mutual marital commitment as a new cognitive process of belongingness to each other”. (A 49-yr-old sex therapist)
	**1.2.2. Secure attachment**	“What happens to my marital life if get worse”? I need to feel my existence is valuable for my spouse”. (A 38-yr-old employee woman with infertility problem)
	**1.3. Compassionate conflict resolving**		
	**1.3.1. Situational insight**	“Couple could change this difficult situation as an opportunity for resolving their marital conflicts with more insight”. (52-yr-old psychologist)
	**1.3.2. Acceptance**	“We did not have good marital relationship before COVID-19, but instead of old repetitive aggressive arguments we need to accept our present situation like millions of other people who got infected with COVID-19. We need to resolve our conflicts by talking”. (A 24-yr-old woman with one child)
**2. Skillful relationship**			
	**2.1. Assertiveness in sexual expression**		
	**2.1.1. Sexual rights fulfillment**	“My concern regarding my husband's loyalty to marital life bothers me badly. Could he betray me? He is a hypersexual man. He watches more porn videos and films these days. How he tolerates these days”? (A 30-yr-old newly married housewife)
	**2.1.2. Resolving reproductive** **concerns**	“I have unprotected intercourse, and I got pregnant unintentionally during the corona outbreak. I worry about my baby”. (A 27-yr-old pregnant woman)
	**2.1.3. Digital age and** **technology-assisted sexual** **relationship**	“My husband and I are excited when we do sexual video chat”. (A 34-yr-old hairdresser)
**2. Skillful relationship**			
	**2.2. Self-efficacy in distance physical connectivity**		
	**2.2.1. Imagination of physical** **intimacy**	“When my spouse sends me a kiss, I close my eyes and feel his kindness and love”. (A 40-yr-old teacher with 2 children)
	**2.2.2. Distance lovely** **conversation**	“Couples who continue their lovely relationship, they overcome their avoidance behaviors”. (A 45-yr-old sex therapist)
**3. Synergy**			
	**3.1. Re-framing spirituality closeness**		
	**3.1.1. Spiritual attention**	“In this situation, many couples experience more attention to spiritual issues like more praying for health”. (A 52-yr-old psychologist)
	**3.1.2. Opportunity for** **hopefully**	“Hopefully helps me to fight against corona strongly, and I will use it in other bad situations in my marital life in the future”. (A 32-yr-old employee maser student)
	**3.2. Empowering aesthetic creativity**		
	**3.2.1. Sharing ideas**	“When I got sick, every night, my spouse and me made funny jokes”. (44-yr-old nurse)
	**3.2.2. Encouragement of** **aesthetic art**	“In these days, couples can use their aesthetic abilities, like baking cake and decorating with the name and picture of the wife”. (44-yr-old midwifery counselor)
	**3.3. Management of family function**		
	**3.3.1. Family responsibilities** **and roles**	“Family cooperation is essential for management of daily life affairs”. (A 56-yr-old sex therapist) “In addition to my children and grandchildren, I take care of my elder mother. I got very upset because of my illness”. (A 52-yr-old women)
	**3.3.2. Connectivity network**	“Although I cannot meet anyone, my family and I keep our connectivity. I have more distance contact with my family members via telecommunications apps”. (A 36-yr-old female worker with 3 children)
	**3.4. Prioritizing**		
	**3.4.1. Time adjustment**	“Because more responsibilities of spouses to manage the family affairs, couples may face a problem for devoting enough time with each other, they should learn to adjust their time”. (41-yr-old couple therapist)
	**3.4.2. Reflection**	“I show my feelings to my husband. When he spends more time with me, I forget my corona disease and feel I am the happiest woman in the world”. (A 40-yr-old teacher with 2 children)

## 4. Discussion

The current study findings on framework qualitative content analysis revealed 3 main categories: 1) mutual resilience; 2) skillful relationship; 3) synergy. In one study concerning helping couples in the shadow of COVID-19, 3 keys that can help couples in supporting their relationships were 1) decide, do not slide; 2) make it safe to connect; 3) do your part (8). In a qualitative study, due to the impact of COVID-19 on sexual health, 3 main themes were mentioned 1) clinical focus; 2) remapping relationships; and 3) reframing technology use (4). In a review article, due to COVID-19 and sexuality, strengthening intimacy and good relationship were recommended for physical, mental, and sexual well-being (5). In one study viewpoints concerning “making love in the time, new changes in marital relationships” were highlighted (1). An article conceptual framework, family beliefs and close relationships were stressed (18). In a research intimate relationship in the correlation of sexual behavior change and family function were highlighted in COVID-19 (6). Relationship issues were emphasized as a shared common concept in all the studies. These findings are similar to the second main category (2. skillful relationship in the current study). The first and third main categories (mutual resilience, and synergy) emerged in current qualitative research. The differences in the findings of our results in comparison with previous articles in the aforementioned categories (mutual resilience and synergy) may be related to different study methodology, target population, main outcome variable, and cultural issues.

The first category pertained to mutual resilience. According to the American Psychological Association, psychological resilience is a process of appropriate adjustment facing adversity, trauma, tragedy, family and relationship problems, and severe health problems (19). Better psychological resilience can buffer the negative emotions of stress and facilitates better performance (20). Strengthening psychological resilience may help foster positive coping styles to benefit their mental health and psychological well-being. Positive coping strategies are positively correlated with psychological resilience, such as mood control, self, and coping flexibility. In one study the importance of increasing resilience was emphasized during the COVID-19 outbreak (2). How spouses cope with pandemic stress, depends on their personal and relational vulnerabilities and resilience (1). Their previous compatibility, adopted coping strategies, and intimacy, determines this relationship's survival (21). In accordance with those assumptions, emerging subcategories of coping strategies, the importance of a healthy marital relationship, and compassionate conflict resolution may explain how resilience can be better understood and accepted in difficult situations by increasing emotional, psychological, and rational tolerance in couples. Emotion regulation is one of the essential tasks in marital relationships that relates to emotional intelligence (22). Couples in difficult situations must learn to regulate intense emotions (23). During the COVID-19 outbreak, couples need emotional safety, which is the ability to talk and be heard, listen, be accepted, work together as a team, and better communication (5, 8). In a research it was reported that during COVID-19, honesty, self-awareness, kindness, and communication are vital for a relationship's security (5, 9). Couples who experience home isolation try to adjust to their new living situation through compassionate conflict resolution. Use of constructive conflict resolution style means it is necessary to tell couples that marital intimacy does not mean not having problems in life but the ability to adapt to problems and solve them (24). Since couples react to (pandemic) stress, some of them may use isolation conditions as an opportunity to create a romantic relationship (1).

The second category, skillful relationship, referred to problems emerging from the impossibility of sexual and physical contact during illness. New behaviors help couples to be realistic about their problems and to solve them via skillful dialogue, to reach a common opinion about the dispute, such as sexual activities (25, 26). Problem-solving, self-disclosure and empathic response skills, and sexual education are effective ways to promote marital intimacy (27). Sex therapists, educators, and physicians may help clients resolve sexual problems by promoting stress management techniques and increasing emotional support and intimacy (9). One of the essential factors in sexual life is a sexual expression (subcategory assertiveness in a sexual expression). Sexual self-expression refers to the transmission of sexual feelings and desires, which, despite its difficulties, is the best way to recognize the sexual preferences of couples (28). Results of a study showed 45% of participants reported their sexual life affected by the COVID-19 epidemic. Incorrect information and uncertainty about COVID-19 sexual transmission routes, fear of intimacy, safe sexual activity, early psychiatric problems, and sexual disorders have significantly increased (5). However sexual abstinence is the safest way to prevent transmission, but it may not be applicable in all cases (7, 29). Sexual fidelity is another crucial issue in this regard (30). These findings are in line with the present study. Women needed their sexual rights to be respected by their spouses' (sexual rights fulfillment) (1). Findings of a study stressed women's reproductive needs like screening of their mental health, domestic violence, sexual relationships, contraception, and perinatal care (31). Separation during disease can genuinely create more openness and intimacy in some partners. In the contemporary digital age, several choices are available to stay erotically connected to a partner: sex toys, webcam, phone sex, mutual distance sexual relationship, sexting, video call sex, swapping pictures, videos, erotic poems or stories, or talk through a fantasy. Sex therapists, educators, and physicians may help their clients by resolving sexual conflict and increasing emotional support and intimacy (9). These findings are in line with the current study results. Women clearly expressed their need to keep a sexual relationship with their husbands during home isolation (digital age and technology-assisted sexual relationship). A skillful relationship can facilitate physical contact. Physical contact automatically leads to intimacy and closeness between the parties (2). Physical safety is defined as having a relationship free from the fear, threat, or experience of physical harm between partners. It is considered bottom-line safety and plays an essential role in COVID-19 (8). These findings are in line with the present study findings. Most of the participants in this study stated concepts such as expressing alternatives to distance physical intimacy, satisfying marital relationships by talking about each other's bodies and praising each other's non-sexual organs, and romantic conversation. It seems that these perceptions regarding physical intimacy during COVID-19 disease are related to changes in physical contact with other communication skills (imagination of physical intimacy and distance lovely conversation).

Synergy was the third category in the present study. Remarkable marital life events such as stress-related illness may aggravate preexisting marital problems or create new difficulties (18). Isolation time is an invitation to learn to live better together in a more collaborative and synergic way. Innovative activities can promote communication, set boundaries, share tasks, experience leisure activities, and reinvent marital intimacy (5). In line with other studies, participants believed couples need to optimize their creativity to manage different aspects of marital life through synergy like more spirituality (subcategory re-framing spirituality closeness), aesthetic creativity (subcategory empowering aesthetic creativity), family affairs (subcategory family function), and prioritizing for spending enough time with each other (subcategory prioritizing). Regarding subcategory re-framing spirituality closeness should be noted, such as transcendent experiences and beliefs, including values, morals, and spirituality, facilitate family resilience (18, 32). Religious people experience positive support and motivation through praying in the face of marital conflict (33). Subcategory empowering aesthetic creativity referred to people who positively reconstruct their thinking frameworks, have more creativity and adaptability in marital life (34). Couples who evaluate their marital performance positively, use their creativity in dealing with obstacles and applying different solutions (35). Subcategory family function showed family processes, well-being, and marital intimacy can be affected through financial insecurity, job loss, cost of illness treatment, caregiving burden, and confinement-related in shadow of COVID-19 (6, 18). Family functions affect male-female intimacy (5). Mental health and well-being determinants for both families and children are crucial (18). In this hardship situation, couples can talk about changes in some responsibilities (responsibilities for children), in the roles related to occupation, sex, resources management, and their relationships with others to make correct decisions about them (8, 18, 21). Social, and recreational intimacy requires involving the spouse in responsibilities, passing holidays, enjoyable activities, leisure time, and expressing experiences and daily events (10). Paying attention to the family by the husband reduces the woman's worries about the family's daily affairs. When a woman with COVID-19 feels the support of her husband, she realizes her existential value in married life. Also, couple therapists emphasize showing love, kindness, consideration, appreciation, empathy, acceptance, humor, and sharing family happiness (36). Subcategory prioritizing is referred to temporal intimacy that indicates couples spending their daily time with their spouse by intimate activities (10). Spending time with each other is one of the indicators of marital quality. Recommending the couple to have recreation together without the presence of children can help them to use their time in an optimized way. During the outbreak of the COVID-19 crisis, spending enough time with each other provides an excellent atmosphere to join each other (2). In one study the role of interpersonal communication in marital interaction was mentioned (37).

Although the period of home isolation is short in women infected with COVID-19, it has important consequences in various dimensions of marital intimacy, their future well-being, and family function. Overall, it should be emphasized that in the COVID-19 outbreak, “make it safe to connect, do your part, decide, do not slide” are best recommendations for saving marital intimacy (8).

The strengths of current study are considering all aspects of marital intimacy, using Bagarozzi's marital intimacy theory as a well-known scientific theory, using qualitative framework content analysis as scientific method, selecting various participants from different socioeconomic statuses, and participation of key informants in various related fields. Since the study was conducted during the COVID-19 pandemic, it can provide rich data regarding women's experiences in home isolation. The findings of this study can be used in other similar diseases. Face-to-face interviews were impossible because women were infected and isolated at home, which can be mentioned as the study limitation.

## 5. Conclusion

The possibility of COVID-19 recurrence, the multidimensional nature of marital intimacy needs like reproductive health issues, marital responsibility and loyalty, mental health consequences, and family well-being challenges reveal the necessity of reinviting new forms of marital relationships in more collaborative and empathic ways. The study's findings could be used by couples, counselors, psychologists, sex and couple therapists, providers, and health system policymakers to better understand marital intimacy needs during the COVID-19 crisis and in other similar diseases in the future. Findings showed that in times of crisis, the need for counseling is especially important for women and should be considered in their treatment program and care guidelines.

##  Conflict of Interest

The authors declare that there is no conflict of interest.
